# Book Notices

**Published:** 1879-01-20

**Authors:** 


### *BOOK NOTICES.*

REST AND PAIN.—A Course of Lectures on the influence of mechanical and physiological rest in the treatment of accidents and surgical diseases, and the dignostic value of pain, delivered at the Royal College of Surgeons of England in the years of 1860–62, by John Hillton, F. R. S., F. R. C. S., surgeon extraordinary to her majesty the Queen, consulting surgeon to Guy's Hospital, late President of the Royal College of Surgeons of England, professor of anatomy, etc., Edited by W. H. A. Jacobson, F. R. C. S., assistant surgeon to Guy's Hospital. Second Edition: New York, Wm. Wood & Co., Publishers.

This is a volume of 287 pages, neatly gotten up and illustrated with numerous cuts. The lectures are able in style and eminently practical, containing much useful information not found in systematic works on surgery.

NOTES ON THE TREATMENT OF SKIN DISEASES, by Robert Leveing, A. M. M. D., Cantab. F. R. C. P., London; late physician and lecturer to Middlesex Hospital, and physician in charge of the Skin Department. Fourth edition, revised and enlarged.—New York, William Wood, publisher, 27 Great Jones street, 1878.

A snug little volume of 123 pages, containing a condensed view of the Anatomy of the Skin, Etiology, Treatment, Classification, etc., of Skin Diseases, with definition of terms and numerous valuable formulæ.

ESSENTIALS OF CHEMISTRY, INORGANIC AND ORGANIC, FOR THE USE OF STUDENTS IN MEDICINE, by R. A. Willhaus, A. M., M. D., Prof. of Chemistry in University of Vermont, etc.—New York, William Wood & Co., publishers, 1879.

This little work is exceedingly valuable to the medical student, and as furnishing to the physician and student of chemistry a condensed review of the science.

